# Real-Time Detection of Behavioral Anomalies of Older People Using Artificial Intelligence (The 3-PEGASE Study): Protocol for a Real-Life Prospective Trial

**DOI:** 10.2196/14245

**Published:** 2019-11-18

**Authors:** Antoine Piau, Benoit Lepage, Carole Bernon, Marie-Pierre Gleizes, Fati Nourhashemi

**Affiliations:** 1 Gérontopôle University Hospital of Toulouse Toulouse France; 2 UMR 1027 Inserm Université Paul Sabatier Toulouse France; 3 Medical Information Department University Hospital of Toulouse Toulouse France; 4 Toulouse Institute of Computer Science Research Université Paul Sabatier Toulouse France

**Keywords:** frailty, monitoring, sensors, artificial intelligence, older adults, participatory design

## Abstract

**Background:**

Most frail older persons are living at home, and we face difficulties in achieving seamless monitoring to detect adverse health changes. Even more important, this lack of follow-up could have a negative impact on the living choices made by older individuals and their care partners. People could give up their homes for the more reassuring environment of a medicalized living facility. We have developed a low-cost unobtrusive sensor-based solution to trigger automatic alerts in case of an acute event or subtle changes over time. It could facilitate older adults’ follow-up in their own homes, and thus support independent living.

**Objective:**

The primary objective of this prospective open-label study is to evaluate the relevance of the automatic alerts generated by our artificial intelligence–driven monitoring solution as judged by the recipients: older adults, caregivers, and professional support workers. The secondary objective is to evaluate its ability to detect subtle functional and cognitive decline and major medical events.

**Methods:**

The primary outcome will be evaluated for each successive 2-month follow-up period to estimate the progression of our learning algorithm performance over time. In total, 25 frail or disabled participants, aged 75 years and above and living alone in their own homes, will be enrolled for a 6-month follow-up period.

**Results:**

The first phase with 5 participants for a 4-month feasibility period has been completed and the expected completion date for the second phase of the study (20 participants for 6 months) is July 2020.

**Conclusions:**

The originality of our real-life project lies in the choice of the primary outcome and in our user-centered evaluation. We will evaluate the relevance of the alerts and the algorithm performance over time according to the end users. The first-line recipients of the information are the older adults and their care partners rather than health care professionals. Despite the fast pace of electronic health devices development, few studies have addressed the specific everyday needs of older adults and their families.

**Trial Registration:**

ClinicalTrials.gov NCT03484156; https://clinicaltrials.gov/ct2/show/NCT03484156

**International Registered Report Identifier (IRRID):**

PRR1-10.2196/14245

## Introduction

### Background

We are facing an increase in the number of older adults with a high prevalence of functional and cognitive decline [1,2]. Early preventive strategies could stabilize or even prevent this decline [1,3,4]. Most frail older individuals are living at home, and we recognize the difficulties in achieving seamless in-home monitoring for the early detection of subtle health changes over time [1,2]. Clinical assessments are usually performed too far apart and outside the person’s own environment. These evaluations rely on self-reported information affected by recall bias and poor reliability [5]. A follow-up before functional or cognitive impairment could have a positive impact through personalized care plans when symptoms can still be treated.

Technology could potentially help to overcome this shortfall in terms of follow-up by providing continuous sensitive and ecologically valid measures. Several real-life studies confirm the relationship between sensor-based monitoring of physiological parameters and health outcomes [6,7]. The follow-up of health changes over time could also support the living choices made by older individuals and their care partners. Some people abandon their desire for independence in favor of the more reassuring environment of a dedicated living facility much earlier than necessary. Nevertheless, although information and communication technologies have been shown to be effective in many medical situations [8], few solutions are proposed to monitor the intrinsic capabilities of older adults in their own homes [9-11]. Beyond *organ-based* telemonitoring solutions (eg, heart failure or diabetes monitoring), a comprehensive function-based monitoring solution could be beneficial to avoid the overlap and multiplication of technical tools in this complex population.

We hypothesized that a network of low-cost sensors could trigger alerts if an acute and unusual event is detected in activities of daily living—ADL (eg, use of the bathroom at night, followed by several hours of immobility) or subtle changes over time (eg, disorganization in stereotypical habits). Our solution based on nonintrusive sensors could provide relevant information to care partners and health professionals to support the monitoring of older people in their homes, thus promoting independent living.

### Objectives

The primary objective is to evaluate the relevance of the alerts automatically generated by a sensor-based solution and the evolution of the algorithm’s performance over time as judged by the recipients: older adults, care partners, and professional support workers. The secondary objective is to evaluate the ability of this solution to detect functional and cognitive decline and major medical events.

## Methods

### Study Design

**Figure 1:**
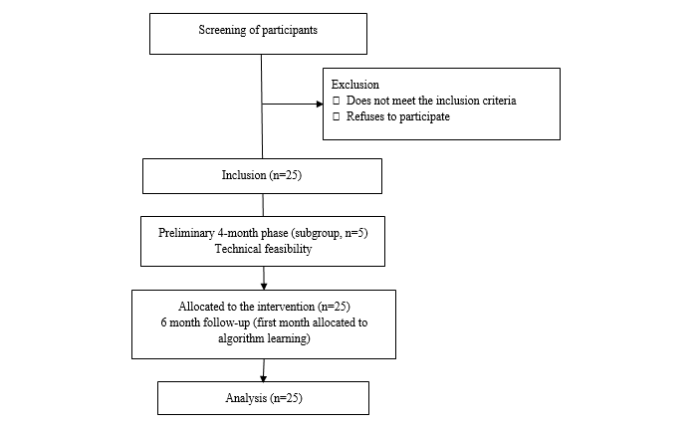
The Standard Protocol Items: Recommendations for Interventional Trials (SPIRIT) flow chart.

This is a prospective open-label study. We will enroll 25 participants for a 6-month follow-up period. To allow for modifications to the solution in the event of technical issues before wider use, the first 5 participants are enrolled for a preliminary 4-month phase before the 6-month regular follow-up period. The enrollment process is spread out over time for the same reason. The flow chart is presented in Figure 1.

### Setting and Participants

All participants must give their written informed consent to take part in the study. Ethical approval was obtained from the Regional Independent Ethics Committee in June 2017 (ID-RCB: 2017-A01002-51).

This solution targets older adults, living with or at risk of functional or cognitive disability and living alone. The detailed inclusion criteria are as follows: aged 75 years or above, living alone at home; frail according to Fried criteria [12] or living with a disability but with an ADL score [13] 3 out of 6 or above; able to walk without help; and provision of written informed consent.

The exclusion criteria are as follows: patient presenting a Mini Mental State Examination (MMSE) <16 out of 30 and without daily care partner support; and under curatorship or guardianship.

Study participants are enrolled through a prescreening procedure on the basis of public administrative records held by several town halls in the region (person aged 75 years or above, living alone at home, and able to walk without help). A clinical research assistant (CRA) then conducts a preinclusion visit by phone. The inclusion visit with the principal investigator (AP) takes place in the Geriatrics Unit at the Toulouse University Hospital.

### Procedure

**Figure 2:**
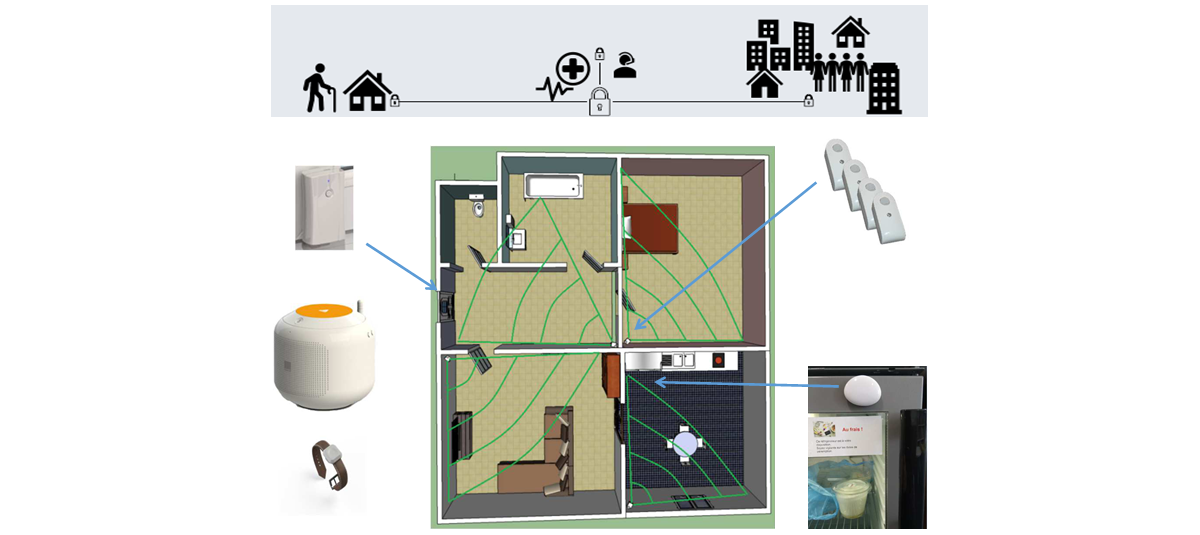
Presentation of the overall solution. In this configuration, the solution comprises 4 passive infrared sensors to monitor activity (bedroom, kitchen, living room, and entrance hall); 1 contact sensor on the main entrance door; 1 cookie sensor on the fridge to monitor door opening; a pendant (or wristband) with a push-button emergency alarm; and a live intercom system (device in the left of the illustration) for direct audio communication with the support center (calling function).

The solution comprises a set of several minimally invasive sensors installed in the individual’s home (see Table 1 and Figure 2). The solution transmits the data to a remote storage server via a *LIVE Intercom* gateway (a commercially available device). Data are then available for remote consultation by authorized users, that is, patients, care partners, or physicians. The *LIVE Intercom* also allows direct audio communication with the support center (call function) and preliminary data processing before 3G transmission to the remote secure health database. The *minimal sensor set* consists of 4 passive infrared (PIR) sensors to monitor activity (bedroom, kitchen, living room, and entrance hall) and 1 contact sensor on the main entrance door. A sensor on the refrigerator door, a pendant (or bracelet) with a push-button emergency alarm, and a detector under the bed will be available to several participants to assess the technical feasibility. The solution is unobtrusive, and no maintenance is required during the study. Technical support is provided in the event of dysfunction.

**Table 1:** Sensor network description.

Device	Number per house	Technology
Wireless motion detector	4	Passive infrared sensor
Wireless door sensor	1	Magnet proximity sensor
Emergency-alert pendant	1	Push button
Intercom with alert button	1	General packet radio services gateway
Wireless bed rest detector	Optional	4 pressure sensors, 1 under each of the feet of the bed
Wireless physical activity tracker	Optional	Accelerometer

The following data will be monitored: presence (yes/no) in specific rooms, for example, rooms associated with eating, bathing, and sleeping; time spent outdoors; total activity inside the home (estimated by the average number of sensor activations per day) [6]; fridge use; and sleeping patterns.

The solution can trigger 2 different types of alerts. The first consists of *conventional alerts*. They are generated by the participants using an alarm push button or through the intercom system. The second type of alert assessed in this project is automatically triggered by our artificial intelligence algorithm after an initial learning phase (eg, gradual change in sleeping pattern). The sensor-based alerts and the use of the Live intercom calling function are both directed to a telecare worker on a nationwide telecare platform (IMA/Serena) all 7 days a week and 24 hours a day. The telecare worker can suggest a range of actions, from direct contact with the older individual to a phone call to the care partner or to emergency services. The first month is dedicated to algorithm learning: all sensors are functional, but no automatic alert is generated from the platform. Activity data relating to sleeping, eating, and time spent outdoors, and so on, are recorded and analyzed. After the first month, there will be sufficient data to detect unusual behaviors and eventfully trigger an alert. The algorithm used to perform real-time detection of behavioral anomalies is described elsewhere [14].

### Follow-Up Procedure

The first 5 participants are enrolled for a total of 10 months (an initial 4-month period followed by a 6-month follow-up period) and the other 20 participants for 6 months. An installation visit will take place in the days following enrollment. A remote follow-up evaluation will be carried out over the phone by a CRA every 2 months after the baseline assessment (see Table 2). The last visit will be conducted by the CRA in the participant’s home after 6 months. For the first 5 participants enrolled in the study, the baseline for data analysis is defined as the beginning of the 6-month follow-up period at the end of the first preliminary 4-month period which is dedicated to technical feasibility. The proper regulatory and ethical conduct of the study is monitored by a clinical research technician, acting on behalf of the University Hospital.

**Table 2:** Data collection.

Variables	Preinclusion (CRA^a^, in-person)	Baseline T0 (investigator)	T2 months (CRA, phone)	T4 months (CRA, phone)	T6 months (CRA, in-home visit)
Informed consent	✓	✓	—^b^	—	—
Sociodemographic data	—	✓	—	—	—
ADL^c^, IADL^d^, MMSE^e^	—	✓	—	—	✓
Frailty criteria, SPPB^f^	—	✓	—	—	✓
Acceptability questionnaire, EQ5D^g^	—	✓	✓	✓	✓
Major medical events	—	✓	✓	✓	✓
Sensor data	—	Continuous measures	Continuous measures	Continuous measures	Continuous measures

^a^CRA: clinical research assistant.

^b^Data not collected.

^c^ADL: activities of daily living.

^d^IADL: instrumental activities of daily living.

^e^MMSE: mini mental state evaluation.

^f^SPPB: short physical performance battery.

^g^EQ5D-3L: EuroQol 5D score to describe and value health, quality of life questionnaire.

### Study Measures

The primary aim is to assess the sensitivity and specificity of the solution, defined as the ability to trigger alerts deemed relevant by the recipients: older adults, care partners, and professional support workers. To this end, both conventional and automatic alerts will be recorded during the follow-up period. Following each alert, the CRA gathers subjective feedback from the participant (for automatic alerts only), the care partner, and the telecare worker: *Was it relevant to alert you?* The alerts are also described (number, time of the day, duration of the communication if a communication is established, subject of the call in case of a conventional alert, and solutions proposed).

The secondary aim is to evaluate the ability of the system to detect a functional or cognitive decline and any major event. All major medical events, defined as any event resulting in in-home physician or paramedic intervention, or a call to the emergency services, are collected retrospectively by the CRA every 2 months (by phone).

Sociodemographic and health data are collected at baseline. The participants’ cognitive and functional parameters are assessed at baseline and at the end of follow-up: MMSE (ranging from 0 to 30; the greater the score, the greater the global cognition) [15]; ADL (ranging from 0 to 6, the greater the score; the greater the functional autonomy in daily life) [13]; Instrumental activities of daily living (ranging from 0 to 8; the greater the score, the greater the functional autonomy in daily life, eg, ability to use the telephone) [16]; Cardiovascular Health Study frailty index criteria (unintentional weight loss, self-reported exhaustion, weakness, slow walking speed, and low physical activity) [12]; Short Physical Performance Battery (consisting of a balance test, a 3-m walking test, and a 5 chair-rises test; score ranging from 0 to 12 with 12 indicating the highest degree of functioning) [17].

The acceptability questionnaire is adapted from the Quebec User Evaluation of Satisfaction with Assistive Technology Scale (degree of satisfaction with technology ranging from 1=not satisfactory at all to 5=very satisfactory) [18]; EuroQol questionnaire to describe and assess health (EuroQol questionnaire 5 dimensions, each comprising 3 levels, are summarized into an index ranging from −0.59 to 1, with 1 indicating full health) [19]; and major medical events. Adverse events and their potential imputability to the monitoring procedure are collected by the CRA throughout the study.

### Sample Size

To the best of our knowledge, no comparable study has evaluated a similar primary endpoint in this population. Therefore, a formal sample size calculation could not be carried out. However, we can expect more than 1 alert per individual during the 6-month period. The predictive value calculations (estimation of legitimate alerts) will be based on numbers over 25, a 95% CI accuracy of at least ±20%. As an example, if a participant triggers 2 alerts on average during the study, with half of them rated as valid, a 95% CI close to 35.5% to 64.5% can be expected.

### Statistical Analysis

The main analysis will be an intent-to-treat analysis. The quantitative outcomes will be estimated with 95% CI. Quantitative variables will be expressed by mean values and standard deviations. The 6-month follow-up period for the 25 participants will allow us to record the number and frequency of alerts generated by the solution. A total of 3 positive predictive value (PPV) calculations (estimation of legitimate alerts) will be done (95% CI, thanks to end users’ feedback for the overall follow-up period and for each 2-month follow-up period: 0-2 months, 2-4 months, and 4-6 months) to estimate the progression of algorithm performance. For the secondary objective, we will also analyze these 3 periods to evaluate the performance of the automatic alerts in detecting changes in functional or cognitive autonomy or major events (sensitivity, specificity, predictive value, and receiver operating characteristic [ROC] curves). Alerts will be addressed with both a binary approach (no alert vs 1 alert to estimate sensibility, specificity, PPV, and negative predictive value) and a continuous approach (number of alerts over the period to plot an ROC graph and estimate the area-under-the-curve). The Department of Epidemiology at our University Hospital will conduct statistical analyses using SAS version 9.4 (SAS Institute, Inc).

## Results

The trial was registered with ClinicalTrials.gov (NCT03484156) on March 30, 2018. The enrollment process is spread out to allow for changes to the system in the event of technical problems.

The first phase with 5 participants (4-month feasibility period) has been completed and the expected completion date for the second phase of the study (20 participants for 6 months) is July 2020.

## Discussion

### Strengths and Limitations of Our Study

Older adults express the desire to live autonomously in their own homes. Clinicians, on the other hand, have difficulty monitoring the functional and cognitive autonomy of seniors over time because of the limitations associated with in-person measurements and self-reported data [5]. To date, despite the rapid pace at which research projects and commercial electronic health devices are developing, few solutions really meet these daily needs. We believe that, following a learning phase, our low-cost unobtrusive solution could trigger alerts if the sensors were to detect an acute, unusual event or a subtle change in everyday habits over time. This solution could provide relevant information to care partners and health professionals to support patient follow-up. The originality of our real-life project lies in the choice of the primary outcome and in our user-centered evaluation rather than in the technical specifications. We will evaluate the relevance of the alerts according to the end users and the progression of our algorithm performance over time. One of the main obstacles to the wide dissemination of alert systems is the low acceptability to end users (eg, false positive alarms) and the difficulty of integrating this approach into a complex and overburdened health care system. In our study, the primary endpoint is determined by the end users themselves. We strive to go beyond traditional medical event considerations such as severe falls, minor falls and minor events, and major events, which do not always make sense for older people. The strengths of our monitoring and support solution can be summarized in several points: it meets the needs of older people living alone; it includes an end user assessment; it is a nonintrusive solution; we use inexpensive sensors without heavy maintenance; and finally, the learning algorithm should increase the solution’s performance over time based on the end user feedback.

Concerning the potential limitations, we plan to analyze the performance of automatic alerts in detecting major events and changes in participants' autonomy (to calculate the number of subjects to be included for a future study). Although our population is at high risk, it is likely that the duration of follow-up is insufficient, particularly for autonomy loss. However, our center implements other studies in comparable populations on which we can rely for such a sample size calculation.

### Our Study in the Context of Previous Research

Several products on the market propose comparable monitoring solutions with very similar technologies [20-22]. However, we were unable to find objective evaluation reports supporting their advertised performance. A review of technologies for monitoring seniors’ home activities identified 5 main types of promising surveillance technologies: PIR motion detectors, worn body detectors, pressure sensors, video surveillance, and sound recognition. This area of research is not totally mature, and most studies did not take place in real-life settings [23]. In a previous study, Franco et al obtained interesting results by recording the electrical activity of 13 subjects monitored for a period of 6 months [24]. The results highlighted the possibility of differentiating between daily and nocturnal activities, and of calculating the probability of having eaten, bathed, or used the toilet with acceptable accuracy. Another study, conducted by Stucki et al, evaluated a nonintrusive, assistive technology system that recognizes and classifies ADL, thanks to passive sensors in each room (20 days, 10 healthy participants, mean age 49 years) with good sensitivity and specificity [25]. Urwyler et al also investigated the behavior of 20 participants using an unobtrusive wireless sensor network for 20 consecutive days. Differences in ADL regimens were significant between healthy controls and patients with dementia [26]. Few academic studies addressed the specific everyday needs of older adults and their families using such a *bottom-up* approach. We think that our study complements previous works.

### Conclusions and Perspectives

Our project brings together partners from the fields of health, technology, industry, and health insurance to develop a relevant but also economically sustainable solution. This is an opportunity for each partner to test the option of integrating such an innovative network into its current practices. Retrospective correlations will be used in this longitudinal study, which justifies further research to prospectively demonstrate the true predictive value of our algorithm.

## Abbreviations

ADL: activities of daily living
CRA: clinical research assistant
MMSE: mini mental state evaluation
PIR: passive infrared
PPV: positive predictive value
ROC: receiver operating characteristic
